# Mammoplasty Using Modified Superomedial Pedicle Technique in Severely Macromastia and Ptotic Breasts

**DOI:** 10.1155/2024/7635485

**Published:** 2024-02-15

**Authors:** Engin Selamioğlu, Özgür Agdoğan

**Affiliations:** ^1^Haliç University, Department of Plastic Reconstructive and Aesthetic Surgery, İstanbul, Türkiye; ^2^Private Clinic, Plastic Reconstructive and Aesthetic Surgery, İstanbul, Türkiye

## Abstract

Mammoplasty is a surgery commonly used for macromastia. Many mammoplasty techniques are described, all with their specific pros and cons. However, the concern to avoid serious complications sometimes takes precedence, and the ideal result cannot be. For macromastia and severely ptotic breasts, usually the free nipple-areolar complex (NAC) mammoplasty technique is implemented. The results, however, may only be completely satisfactory regarding cosmetics. Loss of NAC, poor appearance, flabbiness, flattening, and ptosis are among the disadvantages of this technique. This study aimed to present the results of mammoplasty employing the superomedial pedicle technique without interrupting a macromastia central base with a pedicle length of 8 to 18 cm. According to the literature, many plastic surgeons recommend the free NAC rather than the pedicle technique because of the high complication rates in mammoplasties planned for highly ptotic breasts and macromastia. On the other hand, many free NAC techniques and their modifications with pedicle mammoplasty are described. The general conviction is that a standard method, protocol, or technique good for all patients does not exist. Our results are more acceptable both cosmetically and physiologically. Therefore, the superomedial pedicle technique can be modified to achieve ideal results where free NAC mammoplasty is considered for severe macromastia and ptotic patients. This combined method contributes to the viability of NAC by increasing blood supply to breast tissue and providing an ideal breast appearance.

## 1. Introduction

Even though the etiology of breast hypertrophy is associated with factors such as hormonal and genetic transmission, macromastia develops due to hypertrophy of the hormone-sensitive tissue and glandular epithelium in the breast [1]. On the other hand, breasts in this condition in adolescence constitute gigantomastia. Besides cosmetic problems, severe macromastia and ptotic breasts may cause intense back, neck, and shoulder pain by disrupting body posture [2]. Hygiene problems and dermatitis, bad smells, and fungal infections under the breasts in women frequently accompany chronic and severe pain. Complaints such as pain and numbness in the fourth and fifth fingers due to pressure on the nerves subject to the innervation of nervus ulnaris in the brachial plexus and sleep disorders at night are less frequently reported [3].

It is of utmost importance to maintain the sensitivity of the nipple-areolar complex (NAC) and average circulation in the surrounding tissues in a breast reduction surgery [4–6].

In-depth knowledge of breast anatomy promotes a better understanding of the pros and cons of all mammoplasty techniques and contributes to the safe implementation of the method of choice. Breast tissue has a rich blood supply network [7, 8]. The lateral thoracic artery and internal mammary artery comprise the primary arterial source of NAC [9]. Besides supplying the main vascularity of breasts, the internal mammarian artery contributes to the blood supply of the superior or superomedial pedicle by the branches of the second and third intercostal arteries [10]. Venous drainage of the breast follows the pattern of arterial distribution.

All mammoplasty techniques are based on securing the viability of NAC, except for the free nipple graft technique. The pedicle is composed of glandular tissue or glandular tissue and de-epithelized dermis. Dermis is considered to contribute to venous circulation rather than arterial circulation.

Many breast reduction techniques and modifications are described, including the free nipple, wise pattern, bipedicle, inferior pedicle, vertical pedicle, medial pedicle, superomedial pedicle, superior pedicle, and septal pedicle [11]. Breast reduction with the free nipple graft technique described by Thorek in 1922 is one of the most common techniques for extreme macromastia [12]. Schwarzman was the first to describe the method of moving the NAC on a dermoglandular flap [3, 13]. Later, numerous modifications of the method based on dermoglandular pedicles were described.

NAC was moved over a dermoglandular flap to shape the breast for both functional and ideal aesthetic results. Many of the described techniques have their peculiar pros and cons. The main complications observed include partial or total NAC necrosis, hematoma, hemorrhage, seroma, opening and infection of the wound, cellulitis, reduced or completely lost sensation, and pathological scar.

The main downsides of the free NAC method described by Thorek are poor appearance, flabbiness, flattening, ptosis, loss of sensation, hypopigmentation with discoloration, trouble at breastfeeding, and partial or total nipple loss. Mammoplasty with the pedicle technique on severe macromastia and ptotic breasts always has the risk of NAC necrosis associated with circulatory failure because of the pedicle length and wide tissue resection. Therefore, the free NAC technique is preferred for hypertrophic breasts.

The incidence of partial or total necrosis of the NAC varies in large series; however, it is reported to be between 0.4 and 6% [2]. Despite the complication rates mentioned so far, surgery is still the gold standard.

In this study, we present the results of mammoplasty using the superomedial pedicle technique without disconnecting the flap from the central base on severely ptotic (type-2 and type-3 NAC ptosis) and macromastic breasts with a pedicle length of 8 to 18 cm. In severe macromastia and ptotic breasts, it is to obtain a more ideal and cosmetic breast appearance with the pedicled technique instead of the free NAC graft, which is preferred due to the risk of NAC circulatory failure.

## 2. Material and Method

This study was approved by the Non-Interventional Clinical Research Ethics Committee of Tekirdağ Namık Kemal University, Turkey, in April 2019 (No: 46048792-050.01.04-E.21417). In the preoperative period, mammography or breast ultrasonography (USG) was performed for all patients over 40. This technique transposes the NAC nipple-areola complex through a superomedial dermoglandular flap. In addition to the classical superomedial technique, the flap is not entirely disconnected from the central base.

Patients aged 20–65 years, macromastia with type 2-3 ptosis, and patients with ptosis between 8 and 18 cm from the normal NAC were included in the study. Patients with ptosis less than 8 cm and patients with type 1 ptosis were considered contraindicated for this technique. Patients in whom NAC was difficult to move to its new location as a flap and NAC was planned as a free graft were considered indications for this technique.

**Figure 1:**
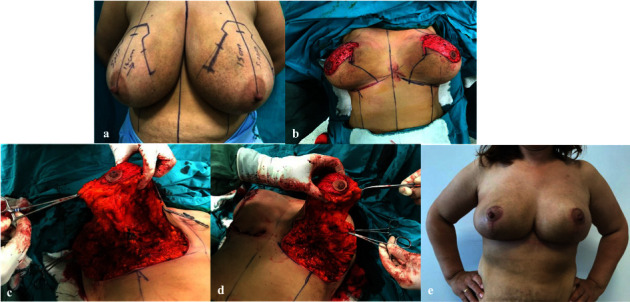
(a) Planning and drawings were made according to the technique. (b) De-epitelized of flap skin. (c) Right NAC flap lifting and base attachment. (d) Left NAC flap lifting and base attachment. (e) Postoperative sixth-month and inverse T scar.

First, plans and drawings were made concerning the technique (Figure 1(a)). Determining the new location of the NAC was the benchmark. After drawing the midline between the suprasternal cavity and the xiphoid, the midline of the clavicle was found, and a vertical line was drawn downwards. This was the line where the NAC would be located. Then, a line was drawn from the suprasternal cavity over this line with a length of 20 to 23 cm. The intersection point of the two lines was determined as the new location of the NAC. On the inframammarian fold, the sternal-medial distance of the new nipple was determined to be 9–11 cm. Once this drawing was complete, the superomedial pedicle flap containing the NAC was also drawn.

The first phase of the surgical operation was the disepithelization of the skin in the superomedial pedicle flap where the NAC was located (Figure 1(b)). The de-epithelized flap was prepared, taking care not to disconnect it from the base (Figure 1(c)). In this sense, the procedure is similar to the inferior dermoglandular pedicle technique. After the flap was secured, excess skin, fat, and gland tissue were excised. Lateral and medial flaps were also removed. Even though it was not disconnected from the central base, the mobility of the NAC was reasonably good because it was highly ptotic (Figure 1(d)). The NAC was set in its new place. At this point, indicating that the NAC flap did not kink when it was reinserted is essential. A drain was placed following hemostasis. Then, the lateral and medial flaps were put together on the midline to cover the de-epithelized portion of the NAC. Glandular tissues of both flaps were fixed with opposite sutures. This also prevented the ptosis of the transposed NAC flap. The lateral flap was fixed over the midclavicular line with a long-term absorbable suture on the pectoral muscle to avoid lateralization. Subcutaneous and intracutaneous sutures were performed, and the surgery was finalized with an inverted T-shaped suturing line (Figure 1(e)).

## 3. Results

**Figure 2:**
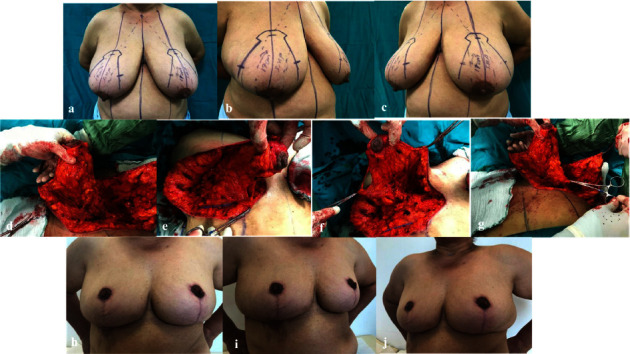
(a) Anterior view of the macromastia and ptotic breast. (b) Right side view of the macromastia and ptotic breast. (c) Left side view of the macromastia and ptotic breast. (d) Right NAC flap elevation. (e) Right NAC flap base attachment. (f) Left NAC flap elevation. (g) Left NAC flap base attachment. (h) Postoperative sixth-month anterior view. (i) Postoperative sixth-month right side view. (j) Postoperative sixth-month left side view.

Between 2013 and 2018, 82 patients who were severely ptotic and macromastia underwent breast reduction surgery in our clinic. In the surgeries, the superomedial pedicle technique in which the NAC was not completely disconnected from the central base was used. No hematoma, hemorrhage, or serious infections were observed. In 7 patients, cut wounds were opened at the conjunction of lateral and medial flaps with the inframammarian fold. Revision of wound lips and resuturation were performed on these patients under local anesthesia. In 1 patient, epidermolysis was found on both nipples, and 3 patients had partial necrosis in unilateral NAC. These were revised and fixed. Nearly ideal NAC was obtained in 3 patients. Acceptable minor asymmetries and dog ears were tolerated. Patients did not consider minor asymmetries and minimal dog ears as a problem and expressed satisfaction with the results. The results obtained were ideal both in cosmetic and functional terms. We presented examples from this case series (Figures 2(a)–2(j)). Tables 1 and 2 show the demographic characteristics of our patients and the complications observed. Comorbid diseases were not analyzed separately, as chronic diseases did not directly interfere with our surgical technique. The number of patients was 82; the mean age was 52, the lowest age was 33, and the highest was 69; 32 patients were chronic smokers. Three patients had a history of nonmammary malignancy.

Comorbid diseases were not analyzed separately, as chronic conditions did not directly interfere with our surgical technique.

The majority of the patients applied to us considering that they had aesthetically ideal breasts, and their complaints such as back, shoulder, or neck pain and itching, and the rash disappeared. We aimed for both cosmetically and functionally perfect results despite the extreme ptotic and macromastia appearance. Tissues ranging from 1,500 to 3,500 gr were resected from each breast. Free nipple grafting is generally recommended for senior patients requiring mammoplasty. However, we applied this pedicle technique also in our senior patients.

**Figure 3:**
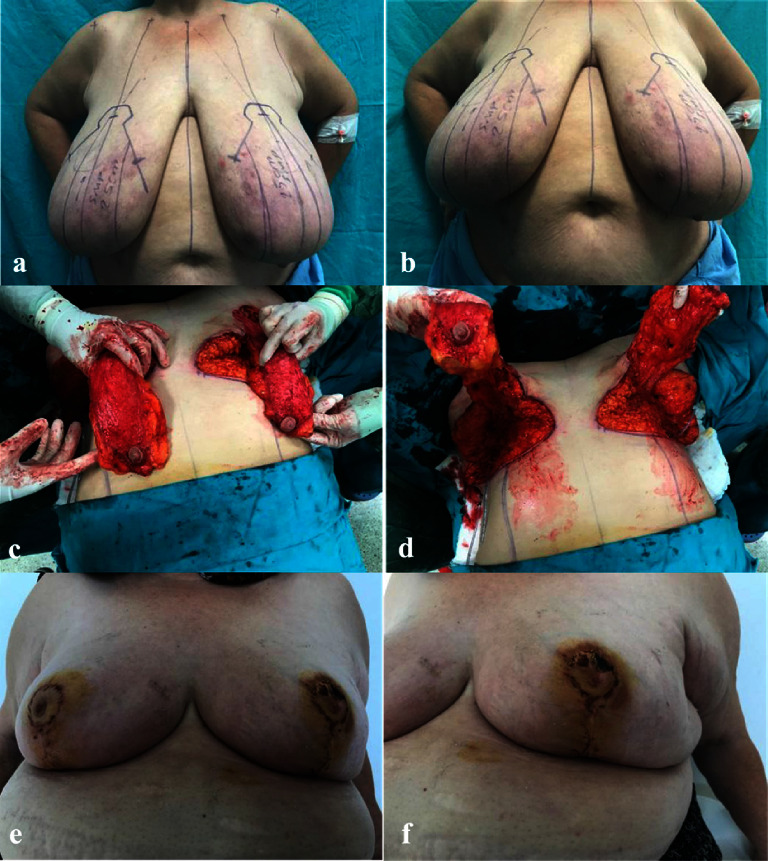
(a) Preoperative drawing. (b) Preoperative technical. (c) Bilateral NAC flap elevation. (d) Bilateral NAC flap base connection. (e) Postoperative sixth-week appearance. (f) Partial superficial loss in the left superior NAC.

Complications that are possible in mammoplasty surgery also apply to this technique. However, necrosis of the NAC is one of the most serious complications. Aiming for the maximum aesthetic and functional results, we minimized this complication by not completely disconnecting the central base when implementing the superomedial technique in our case series, where we had to move NAC for 8 to 18 cm until the new site. We applied this technique to our patients who were ptotic up to 25-26 cm, and no tissue loss was observed (Figures 3(a)–3(f)). As a result, we increased the viability of NAC.

## 4. Discussion

The thickness and length of the pedicle are the most critical limiting factors in NAC transposition [14, 15]. Superior pedicle techniques are reported to be reliable for the transposition of NAC in patients with moderate and large breast hypertrophy and severe ptosis [14, 16]. However, in long pedicled flaps, NAC may kink when being set in its new position, which in turn may impair its circulation. Therefore, as in our study, where long pedicles are the case, the superomedial technique to reset the NAC is more suitable for transposition.

The surgical techniques chosen for breast reduction vary according to several factors, including the NAC pedicle length, new location, age, degree of breast ptosis, and the amount of tissue removed. Standard techniques cannot maintain adequate NAC sensitivity in the postoperative period [17]. Prospective studies show that restoring the nipple-areola sensation gives similar statistical results between superior and inferior pedicle techniques [14, 18]. Therefore, this supports the idea that nipple sensation may not have a disadvantage over the inferior pedicle in our superomedial flap technique. In a retrospective study, no statistically significant relationship was reported between the amount of removed breast tissue and breast sensation [14, 19–21].

Many plastic surgeons do not recommend pedicled mammoplasty methods in breasts where more than 1,000 gr breast tissue excisions are planned due to the high risk of serious complications [2]. For this reason, the main indication of the free nipple-areola technique is patients planned for a breast tissue excision of over 1,000 gr. The presence of systemic diseases, in addition to macromastia, risky NAC viability, obesity, and heavy smoking are other indications [11]. However, our study highly supports the opposite. We applied this technique safely by excising almost 3,500 gr breast tissue from a single breast. Thus, using the pedicled flap technique on our patients with indications for free graft technique, we avoided many possible downsides.

Many pedicle techniques have been defined over the years, and some have been modified as a result of research. However, there is no single technique ideal for shaping breasts of all sizes, and no technique is superior to another [2, 22].

Karsidag et al. used a free nipple graft with the vertical technique for the reduction of highly hypertrophic breasts and reported obtaining sufficiently projected and full breasts [12]. They also obtained durable results in the long run due to the parenchymal sutures they used in the pectoral fascia [12]. Their study supports our technique, namely, fixing the lateral flap on the pectoral fascia with sutures. Thus, it resulted in a more projected breast shape that was not lateralized to the axilla.

Abramo published an alternative technique in which NAC was moved as a superior vertical dermal pedicle up to 7–14 cm on patients with severe breast hypertrophy and ptosis [14]. His study did not include glandular tissue and reported success in sensation [14]. However, his study and technique do not contribute sufficiently to the blood supply in the NAC flap for advanced macromastia and ptotic patients with extensive tissue excision as in our cases.

In their breast-reducing surgeries and study on the inferior pedicle technique, Yılmaz et al. reported the vertical mammoplasty technique could be applied only in cases that required moderate breast reduction. While it was possible to remove 1,000–1,500 gr of tissue with the vertical breast reduction technique, in their study with the inferior pedicle technique, they could easily resect up to 2,500 gr of tissue without any complications [3]. Moreover, they added that using the flap technique in cases requiring more than 2,500 gr resection was impossible, thus recommending the free nipple technique [3]. In our study, in cases of mammoplasty with a resection of over 2,500 gr, the superomedial pedicle flap technique was successfully performed without disconnecting the central base. Therefore, it is worth noting that its use can replace all three methods.

Al-Shaham studied pedicle viability to determine whether to switch to a free breast graft. In mammoplasty operations planned with an inferior pedicle, upon observing circulatory failure in the nipple-areola, they switched to this technique at the expense of extending the operation by 1 or 1.5 hours [2]. In none of our cases, a switch to a free nipple graft technique was considered or required during or after surgery.

In a study performed by Costa et al., reduction mammoplasty with superior medial pedicle technique was performed on highly hypertrophic breasts [20]. They kept the pedicle thickness between 0.8 and 1.5 cm, included only those with a pedicle length of 12–21 cm, and resected tissue ranging from 750 to 3.000 gr from each breast [20]. They reported that mammoplasty with the superior medial pedicle technique was safe and appropriate for highly hypertrophic breasts. Furthermore, they stated that lateral fullness was reduced with this technique [20]. The difference between our study and Costa et al. is that they sacrificed the central base connection and kept the pedicle thin. They also reported that the superomedial pedicle technique, which aims to reduce breast fullness, was more advantageous for transposing the long pedicle and shaping the breast [20, 23]. Comfortable and safe transposition of the pedicle, easy breast shaping, and reduction of lateral fullness were among the results of our study as well. Therefore, they support our technique.

In their study of the thinning of the medial pedicle in reduction mammoplasty, Aboelatta and Aboelatta reported that the medial pedicle could be removed in an ultra-thin fashion without compromising the viability of NAC as long as the patient did not have diabetes. They concluded that this modification contributed to the increase in the flexibility of the medial pedicle, especially in patients requiring extensive reduction [7]. Our study concluded that we could achieve the same flexibility without disconnecting the central base and moving NAC.

Aboelatta and Aboelatta also provided detailed information on the blood supply in breasts. They concluded that the blood supply of the breast included six different locations [7]. In this sense, they stated that NAC received the two most basic vascular supports with the performances from the internal mammarian artery at the 2nd to fourth intercostal spaces and the branches supplied by the lateral thoracic artery at the third and fourth intercostal spaces [7]. In the periareolar region, the branches of the internal mammarian artery and those of the lateral thoracic artery provide an extensive plexus network through anastomosis [7]. In this regard, in our cases, the blood supply of the pedicle was maintained at the highest level to prevent any NAC loss.

In mammoplasty operations, shaping the gland through sutures and fixing it on the pectoral fascia to reshape is part of the techniques by Marchac, DeOlarte, and Lejour [24]. Lassus and Menke did not consider it essential to reshape the breast with deep glandular sutures due to the risk of tissue necrosis through the disruption of vascularization in the glandular and fatty tissue [24]. Würingers septum-based pedicle has also made substantial contributions to the field. Besides, it is solely being used as a source of flap as well as a second supportive perforator added to similar conservative flaps [25]. However, in mammoplasties we conducted with this technique, the fixation of the lateral flap to the pectoral fascia through sutures provided a more aesthetic appearance. It reduced lateral fullness and facilitated the suturing of the glandular tissues of the lateral and medial flaps in the midline. We think that this suture also prevents breast ptosis.

In their study of the surgical treatment in breast hypertrophy, whereby they compared complication rates after large and small tissue excision in the superior pedicle reduction mammoplasties, Fino et al. reported no statistically significant difference between excising more or less than 2,000 gr breast tissue [4]. Likewise, in our cases, the amount of excised tissue and complication rates did not differ significantly.

Our results are more acceptable both in cosmetic and physiological terms. Therefore, we conclude that ideal results can be obtained with a modified superomedial pedicle technique for patients considered for free NAC mammoplasty. Our study included patients with a flap length of 8 to 18 cm. As we did not disconnect the flap from the base, we kept its pedicle thick. On the other hand, lateral and medial flaps were kept thin. This helped achieve central breast fullness while preventing lateral fullness.

Furthermore, the suture fixation of the lateral flap on the pectoral fascia resulted in a more aesthetically satisfying appearance. While reducing lateral fullness, it prevented the breast from flattening. It also facilitated suturing the glandular tissues of the lateral and medial flaps in the midline. Thus, we consider that this suture prevents breast ptosis. Evaluation of the cosmetic result was evaluated with the feedback from the patient satisfaction and our own experiences. There was no negative feedback from the patients in this regard. There was no adverse response from patients regarding the sensitivity of NAC.

Although the technique of our study was similar to the lower dermoglandular pedicle technique, the superomedial technique described the difference. Preserving the base connection is to contribute to the flap and NAC circulation positively. The advantage of this technique is the combination of a superomedial pedicle and a partial central pedicle. So, NAC was modified to increase circulation in advanced ptotic and macromastic breasts. We aimed to increase NAC circulation in advanced ptotic and macromastic breasts by combining the superomedial pedicle and partial central pedicle technique. Thanks to this method, we prevent NAC from being placed as a graft.

The combination of the superomedial technique and the central technique is an alternative to the technique in which NAC is transferred as a free graft, obtaining a better cosmetic result and protecting the milk ducts. In conclusion, we consider that without having any hesitations about the likely complications, mammoplasty can be safely performed by using the superomedial technique in severely ptotic and macromastic breasts without disconnecting the NAC flap from the central base.

## Figures and Tables

**Table 1:** Demographic characteristics of the patients.

Number of patients	82
Average age (lowest-highest)	52.6 (33–69)
8–18 cm NAC ptosis average	15.4
Total amount of tissue excised from both breasts (gram)	2800
Average follow-up period (months)	8.5
Smokers	32
Malignancy history or diagnosis	3

**Table 2:** Complications observed.

Loss of NAC (total/partial)	0/3
Fat necrosis	7
Satisfaction with nipple sensation (very/medium/low)	44/23/9 (6 patients could not be reached or left unanswered)

## Data Availability

The data used to support the findings of this study are included within the article.
